# Sensitive and specific detection of breast cancer lymph node metastasis through dual-modality magnetic particle imaging and fluorescence molecular imaging: a preclinical evaluation

**DOI:** 10.1007/s00259-022-05834-5

**Published:** 2022-05-20

**Authors:** Guorong Wang, Wenzhe Li, Guangyuan Shi, Yu Tian, Lingyan Kong, Ning Ding, Jing Lei, Zhengyu Jin, Jie Tian, Yang Du

**Affiliations:** 1 Department of Radiology, Peking Union Medical College Hospital, Peking Union Medical College & Chinese Academy of Medical Sciences, Beijing, 100730 China; 2 CAS Key Laboratory of Molecular Imaging, Beijing Key Laboratory of Molecular Imaging, the State Key Laboratory of Management and Control for Complex Systems, Institute of Automation, Chinese Academy of Sciences, Beijing, 100190 China; 3 State Key Laboratory of Natural and Biomimetic Drugs, School of Pharmaceutical Sciences, Peking University, Beijing, 100191 China; 4 University of Science and Technology of China, Hefei, 230026 China; 5 Medical School of Chinese PLA, Beijing, 100853 China; 6 University of Chinese Academy of Sciences, Beijing, 100080 China; 7 Beijing Advanced Innovation Center for Big Data-Based Precision Medicine, School of Medicine, Beihang University, Beijing, 100083 China

**Keywords:** Lymph node metastasis, Breast cancer, Magnetic particle imaging (MPI), Fluorescence molecular imaging (FMI), Superparamagnetic iron oxide nanoparticles (SPIOs)

## Abstract

**Purpose:**

A sensitive and specific imaging method to detect metastatic cancer cells in lymph nodes to detect the early-stage breast cancer is still a challenge. The purpose of this study was to investigate a novel breast cancer–targeting and tumour microenvironment ATP-responsive superparamagnetic iron oxide nanoparticles (SPIOs) imaging probe (abbreviated as SPIOs@A-T) that was developed to detect lymph node metastasis through fluorescence molecular imaging (FMI) and magnetic particle imaging (MPI).

**Methods:**

The conjugation of the targeted peptide CREKA and SPIOs was via linker sulfo-SMCC, while the dsDNA-Cy5.5 was modified on SPIOs through the conjugation between maleimide group in sulfo-SMCC and sulfydryl group in dsDNA-Cy5.5. SPIOs@A-T was characterised for its imaging properties, targeting ability and toxicity in vitro. Mice with metastatic lymph node (MLN) of breast cancer were established to evaluate the FMI and MPI imaging strategy in vivo. Healthy mice with normal lymph node (NLN) were used as control group. Histological examination and biosafety evaluation were performed for further assessment.

**Results:**

After injection with SPIOs@A-T, the obvious high fluorescent intensity and MPI signal were observed in MLN group than those in NLN group. FMI can specifically light up MLN using an ATP-responsive fluorescence design. On the other hand, MPI could complement the limitation of imaging depth from FMI and could detect MLN more sensitively. Besides, the biosafety evaluation results showed SPIOs@A-T had no detectable biological toxicity.

**Conclusion:**

SPIOs@A-T imaging probe in combination with FMI and MPI can provide a promising novel method for the precise detection of MLN in vivo.

**Supplementary Information:**

The online version contains supplementary material available at 10.1007/s00259-022-05834-5.

## Introduction

Breast cancer is the most common cancer and the primary cause of cancer mortality in women worldwide [1]. Lymph node metastases (LNMs) are considered the first step toward distant metastases and related to poor outcomes for patients [2, 3]. Therefore, the early, sensitive, and precise detection of LNMs plays a crucial role in guiding breast cancer treatment and improving prognosis. Various clinical imaging approaches, such as magnetic resonance imaging (MRI) and ultrasound (US), have been used for detecting LNMs. However, due to limitations in imaging sensitivity and spatial resolution, these imaging modalities do not achieve sufficient diagnostic sensitivity and specificity [4]. Furthermore, although the benefit-risk ratio of computed tomography (CT) is helpful for clinical application [5], the contrast agent could possibly cause damage to the patients’ renal function [6]. Lymph node biopsy (LNB) is the gold standard for diagnosing breast cancer LNMs [7]. Some radiotracers or fluorescence dye is used for detecting LNMs during LNB [8]. However, LNB is an invasive procedure, is liable to sampling errors, and may cause several adverse effects, such as paraesthesia, lymphoedema, and shoulder abduction deficits [9, 10]. Currently, due to dramatic technological advances, molecular imaging techniques have been used to visualise the breast cancer–related metastatic lymph node (MLN) in vivo before operation, and fluorescence imaging is most frequently used among these techniques. A recent study proposed a pH-amplified nanoparticle for visualising nodal metastases through near-infrared fluorescence imaging (NIRF) [11]. Another study described an application for a dual-targeting nanoparticle, which could differentiate tumour metastases from benign lymph nodes (LNs) by using fluorescence molecular imaging (FMI) and photoacoustic imaging (PAI) [12]. However, because of the tissue scattering and absorption, fluorescence signal still faces light attenuation, resulting in the low tissue depth, and influences the detection sensitivity and accuracy [13]. Furthermore, only 0.7% of the engineered nanoparticles could reach the tumour site on account of the enhanced permeation and retention (EPR) effect, which results in less penetration and accumulation at tumour areas [14, 15]. Thus, an active-targeting, sensitive, and specific imaging method to detect breast cancer LNMs is still a challenge.

ATP is a biomolecule that is essential for cellular energy supply and signal transmission [16]. ATP concentrations are higher in tumour cells than in normal cells. Therefore, the ATP level is distinctly higher in the tumour microenvironment (TME) due to the metabolism and proliferation of tumour cells [17, 18]. This indicates that an ATP-responsive imaging probe can be used for the imaging of the TME [19, 20]. Furthermore, DNA aptamers labelled with fluorescence dyes can exhibit the smart “turn on and off” fluorescence property by sensing specific molecules (e.g. protein) or TME characteristics (e.g. pH or hypoxia) [21–24]. Therefore, FMI with an ATP-responsive aptamer can be applied for the specific detection of the TME.

To resolve the restriction regarding imaging depth of FMI, magnetic particle imaging (MPI), an emerging and novel imaging method, is introduced in this study. Nanoparticles with superparamagnetic properties, magnetic saturation, and nonlinear magnetization curves are considered suitable for MPI [25]. We use superparamagnetic iron oxide nanoparticles (SPIOs), which are also utilised for T2-weighed MRI, as the contrast agent in the present study. Compared with T2-weighed MRI, MPI possesses obvious advantages, such as high sensitivity and a positive signal [25–27]. Importantly, MPI obviates the limitation of imaging depth, and this benefit enables the use of MPI as a promising sensitive method for detecting breast cancer LNMs.

**Scheme 1:**
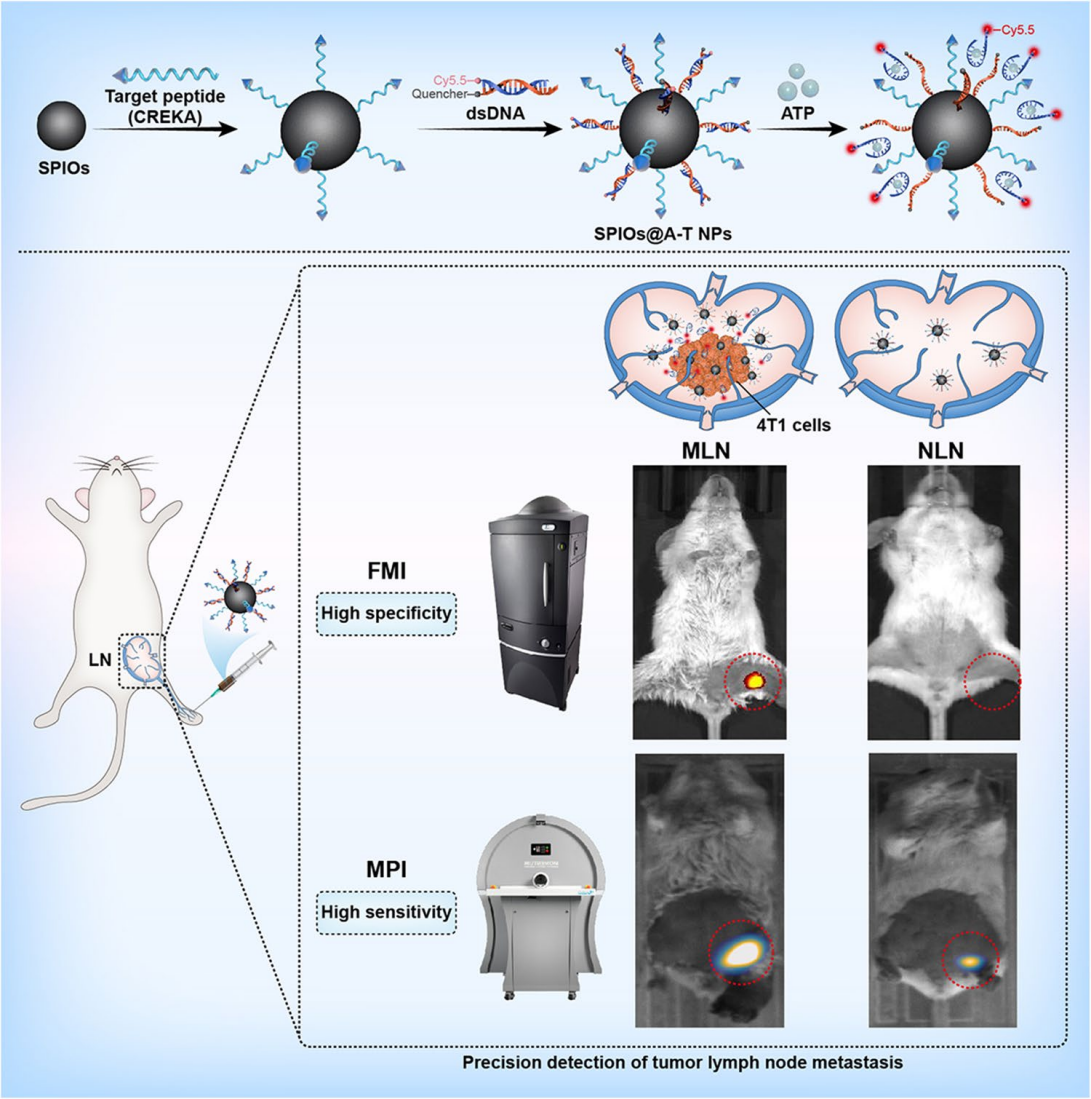
Schematic illustration of synthesis and workflow of the SPIOs@A-T NPs to detect MLN of breast cancer by using dual-modality FMI and MPI to actualise a technique for the specific and sensitive detection of MLN. LN: lymph node, MLN: metastatic lymph node, NLN: normal lymph node, FMI: fluorescence molecular imaging, MPI: magnetic particle imaging

To detect breast cancer LNMs, we designed and evaluated a tumour-targeting and TME ATP-responsive imaging probe, mainly comprising SPIOs conjugated with ATP-responsive aptamer double-stranded DNA-Cy5.5 (dsDNA-Cy5.5) and breast cancer targeting peptide Cys-Arg-Glu-Lys-Ala (CREKA). The CREKA peptide binds to the fibrin–fibronectin complexes that are highly expressed on breast cancer cells and interstitial cells and possesses good tumour-targeting capacity [28–30]. The ATP-responsive fluorescence aptamer has a characteristic that fluorescence only emerges in the presence of ATP. More precisely, the DNA structure designed in our study exists in double-stranded form without ATP, and the fluorescence signal of Cy5.5 is quenched. In the presence of ATP in the tumour environment, the double-stranded structure dissociates and the fluorescent signal of Cy5.5 is released. Therefore, the ATP-responsive design could improve the detection specificity and signal-to-background ratio (SBR) of MLN. Moreover, SPIOs are suitable for MPI-based detection, which can facilitate the visualisation of metastatic cancer cells with high sensitivity and without image-depth limitation. This study aimed to explore the feasibility of the application and the superiority of breast cancer targeting and ATP-responsive nanoparticles (NPs), abbreviated as SPIOs@A-T, to visualise the MLN of breast cancer using FMI and MPI enables a more precision detection of LNMs (Scheme 1).

## Materials and methods

### Materials

The DNA aptamer (Table. S1) was synthesised by Sangon Biotech Co. (Shanghai, China). The target (CREKA) and nontarget peptides (CERAK) were synthesised and purified by Qiangyao Biotech (Suzhou, China). SPIOs were bought from Micromod. The particles are core–shell-structured, and the size is 50 nm. Its magnetization and saturation magnetization are 100 Am^2^/kg iron (*H* = 80 kA/m) and greater than 110 Am^2^/kg iron (*H* = 800 kA/m), respectively. The magnetic nano-flower core is coated with dextran 40 KDa and functionalized with amino-groups.

### Preparation of SPIOs@A-T NPs

The conjugation of the targeted peptide CREKA and SPIOs was via linker sulfo-SMCC, while the dsDNA-Cy5.5 was modified on SPIOs through the cojugation between maleimide group in sulfo-SMCC and sulfydryl group in dsDNA-Cy5.5. Both targeted, ATP-nonresponsive nanoparticles (SPIOs@nA-T NPs) and non-targeted, ATP-responsive nanoparticles (SPIOs@A-nT NPs) were synthesised as control groups. SPIOs@nA-T NPs was realised by modifying Cy5.5-labelled ATP-nonresponsive mutated aptamer. The detailed description is available in the supplementary information.

### ATP-responsive assay

A series of different concentrations of ATP, CTP, GTP, and UTP (0, 0.5, 1, 2, 3, 4, 6, 8, 10 mM) was added to 200 μL buffer solution (5 mM MgCl_2_, 150 mM NaCl, 20 mM HEPES, pH = 7.4) containing 100 nM DNA-responsive aptamer probe (Cy3-dsDNA) in 96-well black plates. The mixtures were slightly shaken in a shaker for 20 min. After that, the mixture solution was imaged using an IVIS Lumina System (PerkinElmer). The same experiments were performed to verify the ATP-responsive ability of SPIOs@A-T NPs and SPIOs@nA-T NPs.

### MPI and MRI imaging properties

SPIOs@A-T NPs were diluted to different concentrations of Fe (0.0625 mM-1 mM) to acquire MPI images (Magnetic Insight Inc, MOMENTUMTM Imager) and MRI (BioSpec 70/20 USR; Bruker, Billerica, MA) signals. SPIOs@A-nT and Vivotrax (Magnetic Insight Inc., USA) were used as two controls. The MPI signal data were further analysed by utilising VivoQuant software (Invicro, Boston, MA). The scanning parameters of MPI were as follows: field of view = 12 × 6 cm, frequency 45 kHz, drive field 16 mT, and magnetic field gradient 6 T m^−1^. The MRI signal was represented by the transverse (T2) relaxation time of all samples. The MRI T2-Turbo RARE sequence was acquired from the following parameters: TR = 3000 ms, TE = 40 ms, field of view = 40 × 40 mm, matrix size = 200 × 200, flip angle = 90°.

### Western blot

Normal lymph node (NLN) and MLN tissues were homogenised and lysed in cold RIPA buffer containing protease inhibitor cocktail. Then, the lysates were centrifuged for 15 min. The supernatants were collected, and the protein concentration was analysed by BCA protein assay (Beijing ComWin Biotech). The samples were incubated with anti-fibronectin polyclonal antibodies (Abcam) or anti-β-actin antibodies at 4 °C overnight. The secondary antibody horseradish peroxidase (HRP)-conjugated goat anti-rabbit IgG (H + L) was used after washing three times in TBST. After incubation for another 1 h and washing, ECL reagents were added, and then Western blot images were captured.

### Cell viability test

The Cell Counting Kit-8 (CCK-8, Solarbio) was used for cell viability test. 4T1 cells were seeded into 96-well plates with 2 × 10^4^ cells in each well. Five parallel wells were set in each group. After incubation for 24 h, cells were exposed to concentrations of SPIOs@A-T, SPIOs@nA-T, and SPIOs@A-nT NPs for another 2 h. Cells with no treatment were used as the controls. Finally, CCK-8 reagent was added, and the absorbance at 450 nm was detected by a VICTOR Nivo Reader (PerkinElmer).

### Confocal laser scanning microscopy

The 2 × 10^5^ 4T1 cells were seeded into 35-mm glass bottom confocal dishes and cultured for 24 h. After that, 500 μL RPMI 1640 medium containing 100 nM SPIOs@A-T, SPIOs@nA-T, and SPIOs@A-nT NPs was added to the confocal dish. After incubation for 2 h, 20 μL of 4% paraformaldehyde was added to fix the cells for 10 min. The cells were washed three times with PBS and then treated with 20 μL DAPI for another 15 min. Finally, the cells were washed thoroughly three times with PBS, followed by imaging using a Leica SP8 STED 3X system.

### Animal model

Five- to 6-week-old female BALB/c mice weighing 18–20 g were purchased from Beijing Vital River Laboratory Animal Technology Co., Ltd. All animal experiments were approved by the Institutional Review Board of Peking Union Medical College Hospital (Permit No: B371-1). 4T1 cells (5 × 10^5^ cells in 50 μL PBS) were injected into the left mammary fat pad and rear footpad of mouse to establish orthotopic breast tumour and breast cancer with metastatic lymph node (MLN) animal model, respectively.

### In vivo FMI

The lymphatic metastasis mouse model mice were intradermally injected with SPIOs@A-T, SPIOs@nA-T, and SPIOs@A-nT NPs in the left hind paws at a ds-DNA probe dose of 25 nmol/kg (*n* = 3). The healthy control group of mice (*n* = 3) was injected with the same dose of SPIOs@A-T NPs. All mice were anaesthetized with isoflurane, and FMI was carried out using an IVIS Lumina System (PerkinElmer). The images were obtained at multiple time points: 0 h, 4 h, 8 h, 12 h, and 24 h. The ex vivo lymph nodes were also imaged after 24 h of in vivo imaging.

### In vivo MPI and MRI

Mice with MLNs were randomly divided into three groups (*n* = 3 mice) according to the injection of different NPs: (1) SPIOs@A-T, (2) SPIOs@A-nT, and (3) Vivotrax. All mice were intradermally injected with the corresponding NPs in the left hind paws at 50 μL of 1 mg/mL Fe. Mice under healthy conditions were considered control mice, which were injected with the same procedures of SPIOs@A-T NPs. MPI (Magnetic Insight Inc, MOMENTUMTM Imager) and MRI (BioSpec 70/20 USR; Bruker, Billerica, MA) were performed at multiple time points.

### Statistical analysis

Data are presented as the mean ± SD and were analysed with GraphPad Prism 8.0 (GraphPad, San Diego, CA). Statistical assessment was carried out by one-way ANOVA within multiple groups or Student’s *t* test for two groups. A *p* value < 0.05 was considered statistically significant.

## Results

### FMI of the ATP-responsive property of SPIOs@A-T NPs

Fluorescence intensity was nearly undetectable in the absence of ATP, but fluorescent signals gradually increased with increasing ATP concentrations (Fig. S1a, S1b). Control groups with CTP, GTP, or UTP exhibited weak responses to the DNA probe even with increasing ATP concentrations. These results suggested that the dsDNA-Cy3 probe can specifically respond to ATP. The fluorescence signal changes showed a linear correlation with the concentration of dsDNA-Cy3 probe (from 0.1 to 2.0 μM) in response to 5 mM ATP (*R*^*2*^ = 0.997, Fig. S1c).

**Fig. 1:**
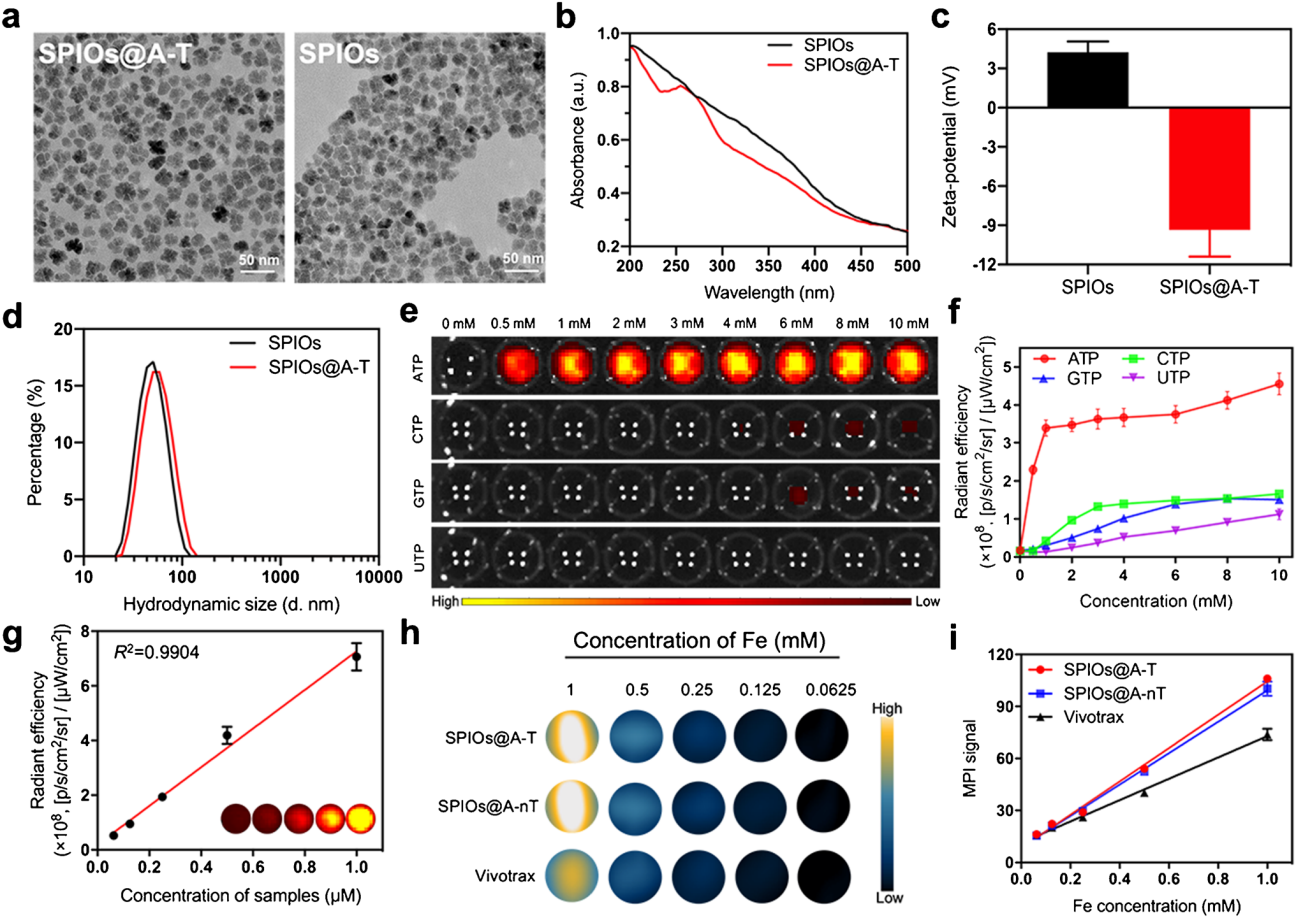
Characterizations of SPIOs@A-T NPs. TEM image of SPIOs@A-T and SPIOs (**a**). UV–vis absorption spectra (**b**), zeta potential (**c**) and hydrodynamic size (**d**) of SPIOs and SPIOs@A-T. Fluorescence imaging of the SPIOs@A-T NPs (**e**) and its corresponding quantitative analysis responding to different nucleoside triphosphates (**f**), [Analytes] = 100 nM. Standard curve of the fluorescence signal over samples with series concentrations of SPIOs@A-T NPs in response to 5 mM ATP (**g**). MPI images of SPIOs@A-T NPs, SPIOs@A-nT NPs and Vivotrax at series of different concentrations (**h**). Plot of MPI signals of all samples (**i**)

The transmission electron microscopy (TEM) image showed that SPIOs@A-T NPs were mono-dispersed and homogeneous, without any obvious change in the shape compared to unmodified SPIOs (Fig. 1a). The ultraviolet–visible (UV–vis) absorption spectra showed that SPIOs@A-T had an absorption peak at 260 nm (Fig. 1b). After modification with CREKA and dsDNA, the zeta potential changed from a positive potential of 4.24 ± 0.83 mV to a negative of − 9.36 ± 2.04 mV (Fig. 1c). The hydrodynamic sizes of SPIOs and SPIOs@A-T were 51.63 ± 16.30 and 58.58 ± 19.34 nm, respectively (Fig. 1d). The abovementioned results verified the successful synthesis of SPIOs@A-T NPs.

As shown in Fig. 1e and 1f, SPIOs@A-T manifested a significant increase in fluorescence signal intensity with increasing ATP concentrations, whereas they showed no change in the response with the addition of CTP, GTP, or UTP. Furthermore, fluorescence intensity maintained a good linear relationship with the SPIOs@A-T concentration at 5 mM ATP (*R*^2^ = 0.990, Fig. 1g). In contrast, SPIOs@nA-T showed a weak fluorescence response to ATP as well as to CTP, GTP, and UTP under the same experimental condition (Fig. S2a, S2b). SPIOs@A-T showed a significantly higher fluorescence intensity than SPIOs@nA-T at the same ATP concentration (****p < 0.0001 in Fig. S2c). Thus, our results revealed that SPIOs@A-T possesses excellent ATP-responsive ability.

### MPI imaging property of SPIOs@A-T NPs

In Fig. 1h, there is no obvious difference of MPI signal between SPIOs@A-T and SPIOs@A-nT, and the quantitative values of MPI signals were both linearly correlated with the Fe concentration (Fig. 1i). Moreover, the MPI signal of SPIOs@A-T was relatively higher than that of commercial Vivotrax at the same Fe concentration (Fig. 1h, 1i), suggesting that SPIOs@A-T possesses good MPI performance. With increasing Fe concentrations, the T2-weighted MRI images gradually darkened (Fig. S3a). Furthermore, the r2 relaxation time of SPIOs@A-T was higher than that of Vivotrax in the same Fe concentration (Fig. S3b). Thus, we successfully developed SPIOs@A-T and proved that it has good ATP-responsive fluorescence and MPI/MRI imaging characteristics.

### Characterisation of cellular targeting and cytotoxicity of SPIOs@A-T NPs

**Fig. 2:**
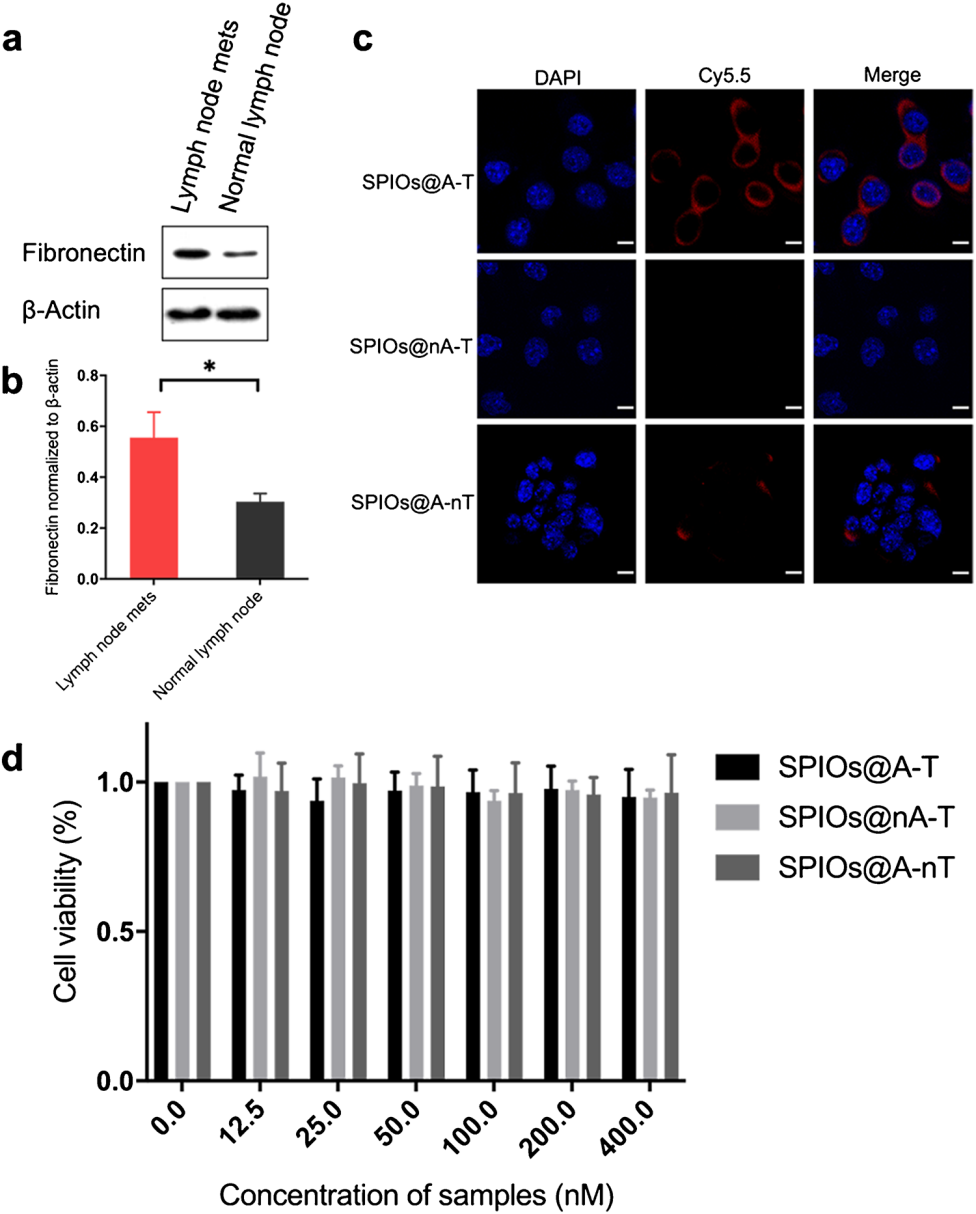
In vitro targeting and cytotoxicity of SPIOs@A-T NPs. **a** The fibronectin protein expression level in lymph node metastases and normal lymph node examined by Western Blot analysis. **b** Densitometric analysis of fibronectin protein expression normalised to that of β-actin. Data represent the mean ± SD, *n* = 3. Mets, metastases. *p < 0.05. **c** Confocal images of 4T1 cells treated with different NPs. Blue fluorescence indicates nuclei staining and red fluorescence indicates corresponding NPs. Scale bars: 20 μm. **d** Cell viability of 4T1 cells treated with different concentration of nanoparticles for 24 h

Western blot data showed that MLNs showed a 1.8-fold higher fibronectin expression compared to NLN (Fig. 2a, 2b, *p < 0.05), suggesting that fibronectin is highly expressed in metastatic tumour cells within LNs.

The confocal images showed that an obvious red fluorescence signal was observed in the SPIOs@A-T group, and the merged images indicated good SPIOs@A-T uptake by 4T1 cells (Fig. 2c). The red fluorescence signals were weak for both SPIOs@nA-T and SPIOs@A-nT due to their non-ATP-responsive and non-targeting properties, respectively (Fig. 2c).

The cytotoxicity analysis demonstrated that cell viability was 90–100% for these three groups, indicating that nanoparticles have no obvious cytotoxicity (Fig. 2d).

### In vivo FMI/MPI dual-modality imaging of orthotopic breast tumour

**Fig. 3:**
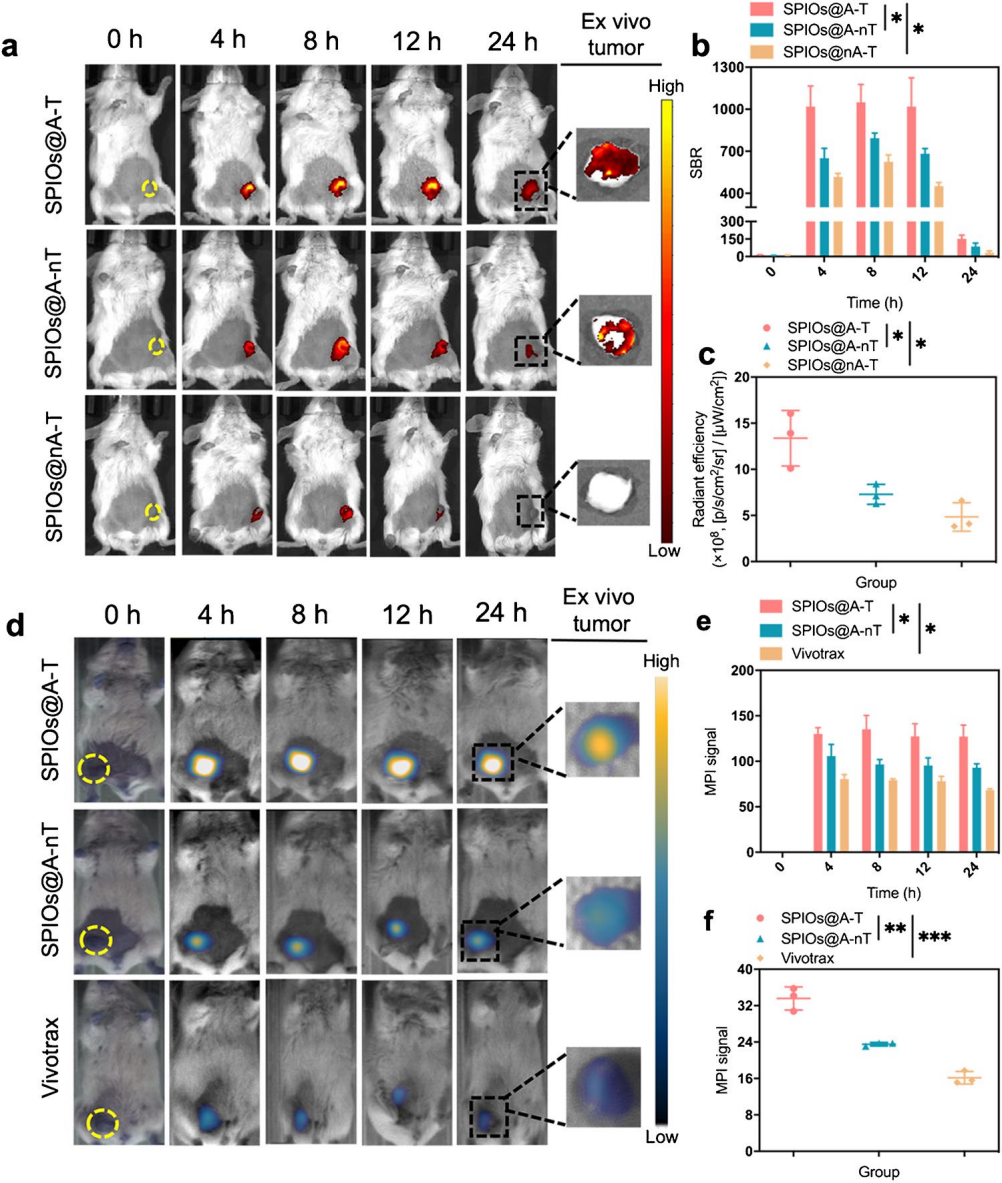
FMI and MPI for 4T1 orthotopic tumour. In vivo and ex vivo (**a**) FMI of 4T1 orthotopic tumours (yellow circle) in mice treated with SPIOs@A-T, SPIOs@A-nT, and SPIOs@nA-T at multiple timepoints after injection (0, 4, 8, 12, and 24 h). The comparison of fluorescence intensity among SPIOs@A-T, SPIOs@A-nT, and SPIOs@nA-T in vivo (**b**) and ex vivo (**c**). *p < 0.05. In vivo and ex vivo (**d**) images from MPI of 4T1 orthotopic tumour (yellow circle) mice treated with SPIOs@A-T and SPIOs@A-nT and Vivotrax at multiple timepoints after injection (0, 4, 8, 12, and 24 h). The comparison of MPI signal values among SPIOs@A-T and SPIOs@A-nT and Vivotrax in vivo (**e**) and ex vivo (**f**). *p < 0.05, **p < 0.01, ***p < 0.001

First, to evaluate the specific and targeted imaging of SPIOs@A-T in vivo, an orthotopic 4T1 breast cancer murine model with small tumours (diameter < 4 mm) [31] was created and imaged after intratumoural injection of SPIO@A-T, SPIOs@A-nT, and SPIOs@nA-T respectively. The results showed that the SPIOs@A-T group showed a higher fluorescence intensity than the non-targeted SPIOs@A-nT for a 24 h post-injection observation period, indicating that, in addition to the EPR effect in vivo, the targeting CREKA peptide could effectively facilitate the targeted binding of nanoparticles to the tumour site (Fig. 3a, 3b). The fluorescence intensity of the SPIOs@A-T group at tumour sites was significantly stronger than that of the SPIOs@nA-T group at different time points after injection, which suggested that SPIOs@A-T nanoparticles could activate fluorescence signalling in the presence of ATP in breast tumours in vivo. Moreover, after the in vivo observation, the tumours were dissected for ex vivo FMI observation (Fig. 3a), and the results suggested that mice injected with SPIOs@A-T exhibited the highest fluorescence signal intensity among the three groups (Fig. 3c).

Furthermore, the MPI was further undertaken to test the feasibility of sensitive imaging for orthotopic breast tumour. As shown in Fig. 3d and 3e, the MPI signal was detected at the tumour site 4 h after the injection of SPIOs@A-T, SPIOs@A-nT, and Vivotrax, and the signal intensity peaked at 8 h after the injection. The MPI signal of SPIOs@A-T was notably higher than those of SPIOs@A-nT and Vivotrax at all time points. The ex vivo tumour images from MPI revealed that mice treated with SPIOs@A-T showed significantly higher signal intensities than other groups (Fig. 3d, 3f).

### In vivo FMI/MPI dual-modality imaging of MLNs of breast cancer

**Fig. 4:**
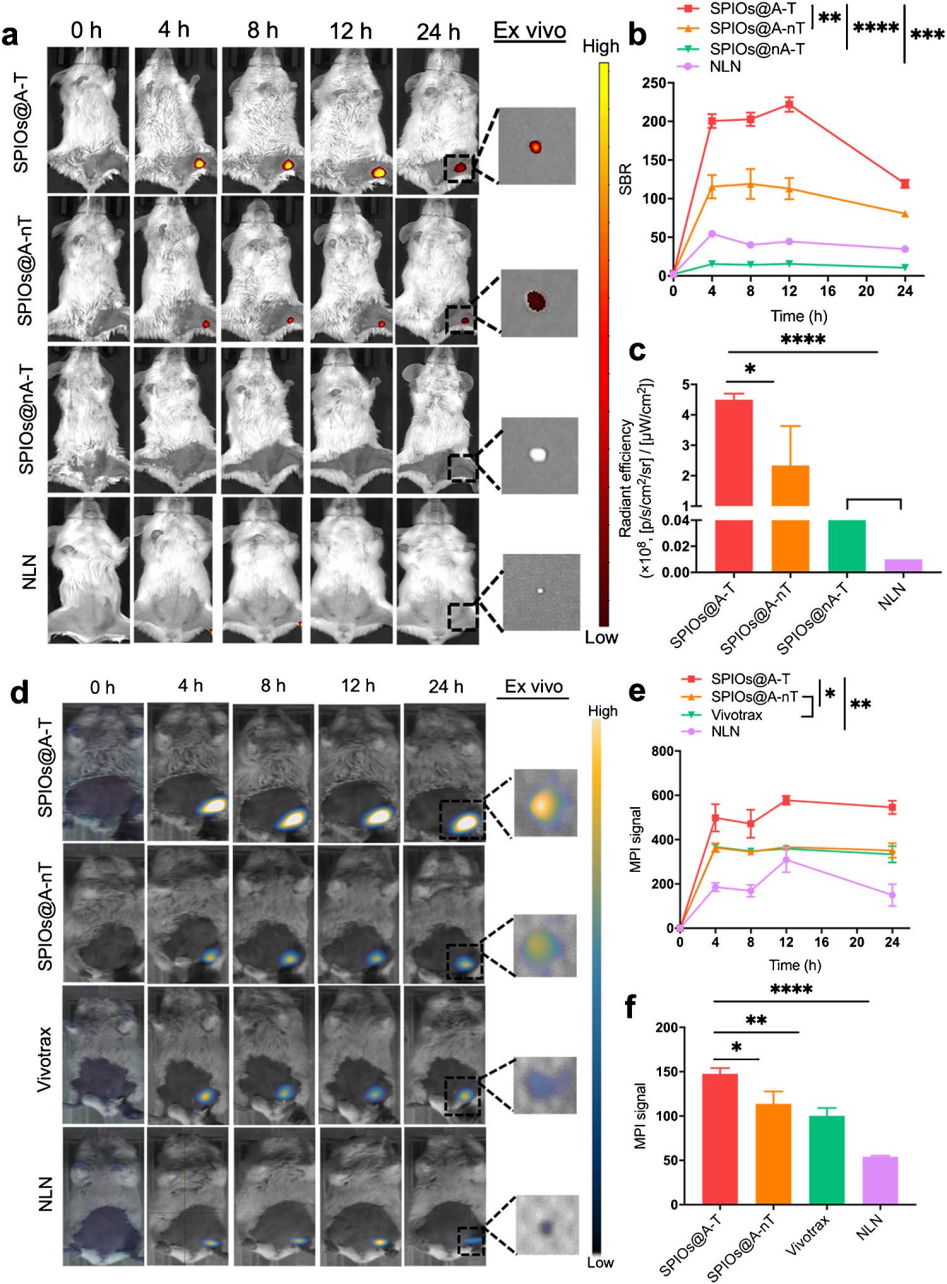
FMI and MPI for lymph nodes. **a** In vivo and ex vivo fluorescence images of MLN treated with SPIOs@A-T, SPIOs@A-nT, and SPIOs@nA-T. NLN treated with SPIOs@A-T is the control group. Images were acquired at multiple timepoints (0, 4, 8, 12, and 24 h) after injection. **b** The comparison of SBR among these groups in vivo. **c** The comparison of fluorescence intensity among these groups ex vivo. *p < 0.05, **p < 0.01, ***p < 0.001, ****p < 0.0001. **d** In vivo and ex vivo images from MPI of MLN treated with SPIOs@A-T and SPIOs@A-nT and Vivotrax. NLN treated with SPIOs@A-T was seen as control group. Images were acquired at multiple timepoints (0, 4, 8, 12, and 24 h). The comparison of MPI signal values among the study groups **e** in vivo and **f** ex vivo. *p < 0.05, **p < 0.01, ****p < 0.0001. MLN, metastatic lymph node; NLN, normal lymph node

After intradermal injection of SPIOs@A-T in MLN mice, the fluorescence intensity augmented notably and peaked at 12 h, and the signal was detectable for 24 h (Fig. 4a). In contrast, only a very weak fluorescence signal was detected in the remaining three groups, suggesting that SPIOs@A-T augment the targeted and specific metastatic breast tumour cell detection capability. Due to the lack of ATP-responsive fluorescence in the TME, mice injected with SPIOs@nA-T presented almost no fluorescence signal at every time point. Similarly, due to the lack of the CREKA targeting peptide, mice injected with SPIOs@A-nT showed a relatively lower signal intensity than those treated with targeted SPIOs@A-T. Quantitative analysis showed that the average SBR of the SPIOs@A-T group was 222.02 ± 9.33, which was 14.3- and 1.96-fold higher than those of the SPIOs@nA-T (15.51 ± 1.22) and SPIOs@A-nT (113.01 ± 13.84) groups, respectively, at 12 h post-injection. To verify the specific detection of SPIOs@A-T for MLNs, the mice with NLNs were utilised as controls. The NLN group did not manifest an observable fluorescence signal, further demonstrating that SPIOs@A-T could specifically detect malignant LN (Fig. 4a). The average SBR of SPIOs@A-T (222.02 ± 9.33) was 4.99-fold higher than that of the group with NLN mice (44.47 ± 3.53) 12 h after FMI in vivo (Fig. 4b). Moreover, 24 h after injection of different NPs, the LN were dissected out for ex vivo FMI. As expected, MLN in the SPIOs@A-T group demonstrated the most obvious fluorescence signal compared to the other two groups. In the SPIOs@A-T group, the corresponding quantitative fluorescence intensity of LNs ex vivo was 4.5 ± 0.2 × 10^8^, which was 16.7- and 1.93-fold higher than that of the SPIOs@nA-T (0.27 ± 0.06 × 10^8^) and SPIOs@A-nT (2.33 ± 1.30 × 10^8^) groups (Fig. 4c). The sizes of MLN and NLN are shown in Fig. S4a. Histological examination of LNs further revealed findings that were consistent with in vivo and ex vivo FMI observation (Fig. S4a; tumour cells in LN are indicated by black arrows).

As shown in Fig. 4d, at 4, 12, and 24 h after the injection, the MPI signal of SPIOs@A-T was localised in the popliteal LN, gradually peaked, and remained stable, respectively. Quantitative assessment at 12 h of the non-targeted SPIOs@A-nT and commercial Vivotrax showed a value of 577.68 ± 19.99 for SPIOs@A-T, which was 1.52- and 1.61-fold stronger than those of SPIOs@A-nT (366.15 ± 9.25) and Vivotrax (359.13 ± 14.03), respectively (Fig. 4e). Moreover, the NLN group was used as the control group. MPI signal values of the SPIOs@A-T group was 1.87-fold higher at 12 h than that of the NLN group due to the specific targeting effect of CREKA (Fig. 4e). After 24 h in vivo observation, the lymph nodes were dissected out for further ex vivo MPI. The results were consistent with that of the in vivo observation (Fig. 4d, 4f). The Prussian blue staining and histology showed more Fe-positive staining in the MLNs of the SPIOs@A-T NPs group compared to other groups (Fig. S4b), which was consistent with the findings of the in vivo observation.

Moreover, T2-weighted MRI provided anatomical structural information of LNs, which exhibited a darkening signal at the left popliteal LN site 2 h post-injection with SPIOs@A-T, and the location was consistent with the FMI and MPI signal areas (Fig. S5).

**Fig. 5:**
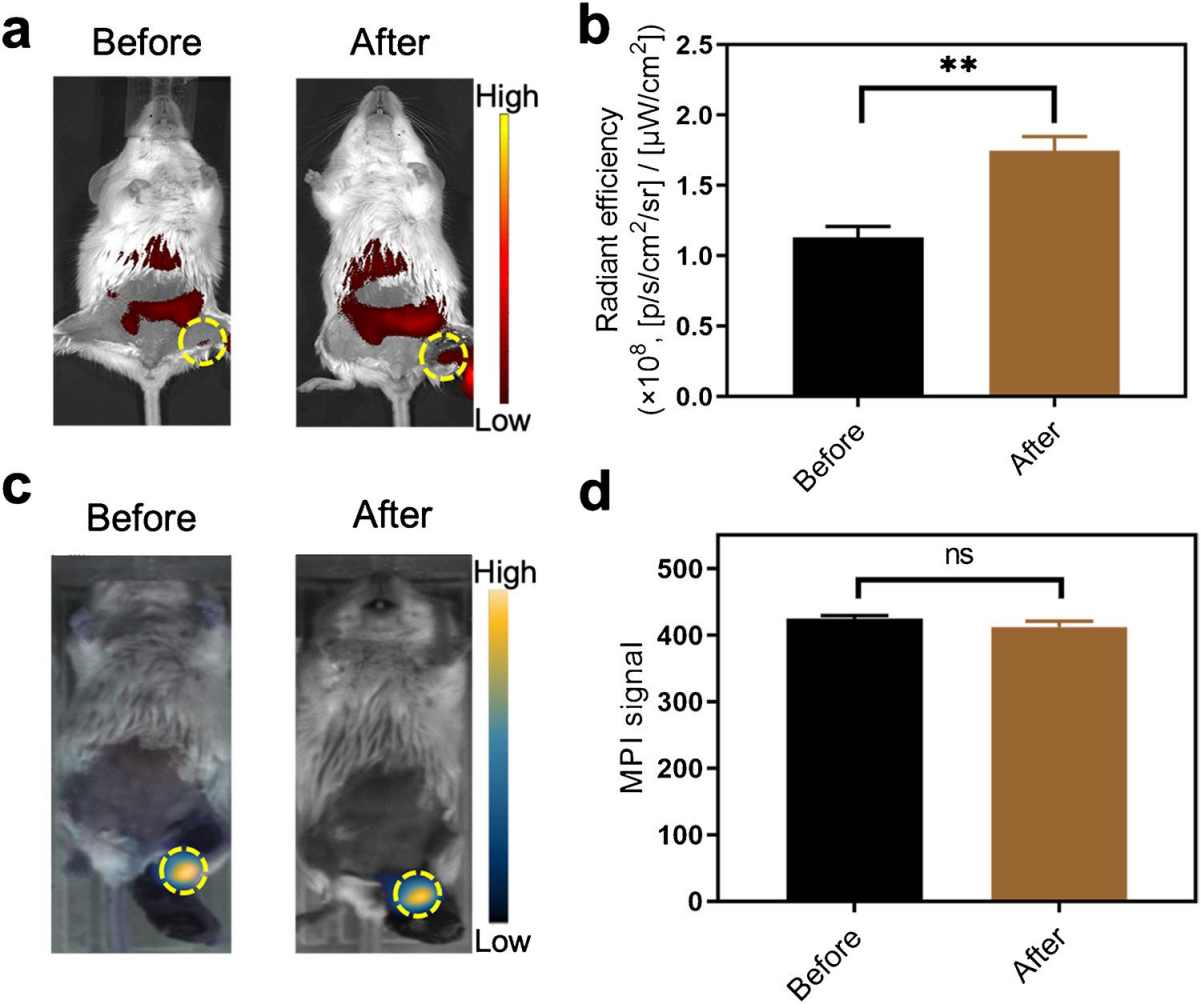
Comparison of the signal-intensity changes of FMI and MPI before and after skin incision. FMI images (**a**) and signal intensity (**b**) of lymph nodes (LNs; yellow circles) before and after skin incision. MPI images (**c**) of LNs (yellow circles) and signal comparison (**d**) before and after skin incision. **p < 0.01. ns, no significant difference

Interestingly, MPI is well known for the superior performance with high sensitivity and the absence of an imaging-depth limitation and, therefore, MPI can be utilised for quantitative analysis. To validate this aspect in our study model, we performed the dual modality imaging scan on the same mice with MLNs both before and after skin incisions. Mice were injected with SPIOs@A-T NPs and scanned at 12 h post-injection. Compared to before skin incision, the fluorescence signal was significantly higher after the skin incision, while there was no remarkable difference for MPI signal between the before and after skin incision evaluations (Fig. 5). This suggests that MPI is a promising method for the detection of metastatic breast cancer cells in LN or even in deep metastases to other organs.

Finally, the immunofluorescence staining was further performed on MLN tissue sections, and the data showed that SPIOs@A-T displayed the strongest fluorescence intensity, compared to SPIOs@nA-T and SPIOs@A-nT. Moreover, SPIOs@A-T specifically bound to metastatic cancer cells, showing merged images (denoted by white arrows). In general, we concluded that SPIOs@A-T NPs can specifically discern and bind to the metastases of breast cancer in LNs (Fig. S6).

### In vivo biosafety assessment of SPIOs@A-T NPs

There were no observable pathological changes for major organs, including the heart, liver, spleen, lungs, and kidneys, in all groups (Fig. S7). In addition, the serum biochemical indicators, including HDL-C as heart function biomarkers, BUN and CREA as kidney function biomarkers, and ALT and AST as liver function biomarkers, were measured (Fig. S8). The data indicated no significant differences between nanoparticle-treated groups and the normal saline group. Thus, SPIOs@A-T, SPIOs@nA-T, and SPIOs@A-nT NPs showed no obvious physical damage in vivo.

## Discussion

Given the importance for early detection of breast cancer LNMs, we developed a novel breast tumour-targeting and smart detection-imaging probe with SPIOs@A-T NPs, which are SPIOs conjugated with the breast tumour-targeting peptide CREKA and an ATP-responsive aptamer. When used in combination with the imaging strengths of FMI and MPI, nodal metastases of breast cancer can be detected with high sensitivity and specificity to differentiate malignant LNs from normal ones. Thus, our study presents a novel and potential imaging method to provide guidance for the detection of breast tumour LNMs in vivo.

Specific and sensitive visualisation of LNMs in early-stage cancer can maximise the benefits of early tumour staging and guidance of treatment selection [32, 33]. We developed a unique ATP-responsive and breast cancer-targeting imaging probe to visualise MLNs in breast cancer in an actively targeted, specific, and sensitive manner. The CREKA peptide was used as the targeting element of NPs, and the results verified that the peptide showed excellent binding ability to metastatic breast cancer cells in LNs, both in vitro and in vivo. Results of both FMI and MPI demonstrated not only that the signal intensity of the targeted SPIOs@A-T is quantitatively stronger than that of non-targeted SPIOs@A-nT, but also that SPIOs@A-T would prolong the retention time of the signal at the LN area. Furthermore, we adopted an ATP-responsive fluorescence element in our nanoprobe to further improve the detection specificity for metastatic cancer cells in LNs. In our in vivo FMI experiment, we found that SPIOs@A-T NPs showed an obvious, more intense fluorescence signal at MLN regions compared to SPIOs@nA-T NPs.

In this study, we introduced a dual-modality imaging method combining FMI and MPI to visualise tumour MLNs. On the one hand, FMI can specifically light up MLNs using an ATP-responsive fluorescence design. Since the 4T1 tumour cells in MLNs show high ATP concentration, which can activate the SPIOs@A-T NPs and release the Cy5.5 fluorescence, hence, the MLNs can be specifically lightened and detected by FMI. The significance of MPI for MLN detection in this experiment is mainly based on its several characteristics, including (1) high sensitivity, which is suitable for the early detection of small metastases; (2) quantitative imaging, the imaging signal is linearly quantitative with SPIOs content; (3) no image-depth limitation compared with FMI; (4) no background interference, which can reduce false-negative and false-positive results; and (5) safety, which does not have radioactivity or biohazards.

Based on the current preliminary studies, we next plan to apply MPI to image MLNs before surgery and use intraoperative fluorescence-image guidance for further identification and precise resection of MLNs. MPI is not limited by tissue depth, and it exhibits positive signal values that are linearly correlated with the concentration of SPIOs. Therefore, by comparing MPI signals before surgery, we could determine the difference of tumour-targeted SPIO uptake between metastatic and non-metastatic lymph nodes and then preliminarily distinguish MLNs from NLNs. FMI shows high spatiotemporal resolution, and hence, it has great potential for intraoperative imaging and navigation [34]. With the specific imaging of MLNs and the high SBR demonstrated by FMI, it can be used for accurate intraoperative identification and guidance of precise MLN resection. Combined with the complementary advantages of MPI and FMI, it is expected to achieve more sensitive detection and accurate excision of MLNs. Moreover, the lymph node tissue of breast cancer patients can be tested by SPIOs@A-T NPs in the future study.

In conclusion, a novel dual-modality molecular imaging probe, SPIOs@A-T NPs, was integrated with two complementary FMI and MPI techniques and manifested the feasibility for diagnostic evaluation to distinguish MLN from NLN in a preclinical breast cancer model. On the one hand, the unique ATP-responsive fluorescence design allows FMI to specifically light up the lymphatic metastasis. On the other hand, MPI can compensate for the limited imaging-depth defect of FMI and to ensure sensitive imaging of metastatic tumour cells. Hence, SPIOs@A-T NPs is expected to be a powerful tool for early detection and accurate diagnosis of MLN in vivo.

## Supplementary Information

Below is the link to the electronic supplementary material.

Supplementary file1 (DOCX 8650 kb)

## Data Availability

The datasets generated during and/or analysed during the current study are available from the corresponding author on reasonable request.
