# Supplementary material for: Sensitive and specific detection of breast cancer lymph node metastasis through dual-modality magnetic particle imaging and fluorescence molecular imaging: a preclinical evaluation
Source: Eur J Nucl Med Mol Imaging. 2022 May 20;49(8):2723–34. doi: 10.1007/s00259-022-05834-5 (PMC9206605; doi:10.1007/s00259-022-05834-5)
Supplement: Supplementary file 1 — Supplementary file1 (DOCX 8650 kb) [file 259_2022_5834_MOESM1_ESM.docx]

**Supplementary information**

**Sensitive and specific detection of breast cancer lymph node metastasis through dual-modality**

**magnetic particle imaging and fluorescence molecular imaging: a preclinical evaluation**

**Authors**

Guorong Wang, Wenzhe Li, Guangyuan Shi, Yu Tian, Lingyan Kong, Ning Ding, Jing Lei, Zhengyu Jin*, Jie Tian*, Yang Du*

**Corresponding authors**

Zhengyu Jin

Affiliation: Department of Radiology, Peking Union Medical College Hospital, Peking Union Medical College & Chinese Academy of Medical Sciences, Beijing 100730, China.

Email address: jin_zhengyu@163.com

Jie Tian

Affiliations: 1 CAS Key Laboratory of Molecular Imaging, Institute of Automation, Chinese Academy of Sciences, Beijing, 100190, China.

2 University of Chinese Academy of Sciences, Beijing 100080, China.

3 Beijing Advanced Innovation Center for Big Data-Based Precision Medicine, School of Medicine, Beihang University, Beijing, 100083, China.

Email address: [jie.tian@ia.ac.cn](mailto:jie.tian@ia.ac.cn)

Yang Du

Affiliations: 1 CAS Key Laboratory of Molecular Imaging, Institute of Automation, Chinese Academy of Sciences, Beijing, 100190, China.

2 University of Chinese Academy of Sciences, Beijing 100080, China.

Email address: yang.du@ia.ac.cn

**Supplementary methods**

**Preparation of SPIOs@A-T NPs**

SPIOs-NH2 solution (10 mg/mL) at an amount of 100 μL was first dispersed in 400 μL HEPES buffer (20 mM, pH = 7.4). After that, they were mixed with 300 μL CREKA (10 mg/mL) and 300 μL sulfo-SMCC (10 mg sulfo-SMCC in 300 μL DMF) solution for 2 h. Then, the CREKA-maleimide-activated SPIOs solution was centrifuged at 1,3000 rpm for 10 min. Subsequently, the reaction mixture was washed and redispersed in 100 μL 1× PBS buffer for the next hybrid double-stranded DNA aptamer (denoted as hApt) conjugation. Second, to acquire hApt, we added 12 μL complementary DNA aptamer (denoted as cApt, 100 μM) and 10 μL thiolated single-stranded DNA aptamer (denoted as HS-Apt, 100 μM) to 76 µL buffer solution (20 mM HEPES, 5 mM MgCl2, 150 mM NaCl). Afterwards, the mixture solution was heated for 5 min at 95 °C, and then slowly cooled to room temperature to obtain hApt. Next, 10 μL TCEP solution was added to hApt for another 1 h, and then the mixture solution was centrifuged using a 3 KDa Amicon Ultra filter to remove the extra TCEP. Then, the supernatant from the 3 KDa Amicon Ultra filter tube was added to the activated SPIOs solution and reacted overnight. Finally, the synthesized SPIOs@A-T NPs were collected under centrifugation at 1,3000 rpm for 10 min, washed three times, and then stored at 4 °C for subsequent use.

**Cell culture**

The murine breast cancer cell line 4T1 was acquired from the American Tissue Culture Center (Manassas, VA, USA). 4T1 cells were cultured in RPMI 1640 containing 10% foetal bovine serum and 1% penicillin/streptomycin at 37 °C with 5% CO_2_.

**Histology and Prussian Blue Staining**

After *in vivo* imaging, the lymph nodes were dissected and preserved in 4% paraformaldehyde for further embedding and sectioning. All lymph node (LN) samples were sectioned with a slice thickness of 4 μm. Haematoxylin and eosin (H&E) staining and Prussian blue staining were utilized to manifest the histological changes and Fe distribution in the tissue, respectively. The tissue slices were scanned by a PANNORAMIC MIDI II scanner (3DHISTECH, Hungary).

**Lymph nodes immunofluorescence staining**

To display the distribution of SPIOs@A-T, SPIOs@nA-T, and SPIOs@A-nT NPs in LNs, mice with lymphatic metastasis were sacrificed 12 h after intradermal injection of the three kinds of tracers. The LNs were dissected and fixed in 4% paraformaldehyde. Then, all tissue samples were wax-embedded and sectioned into four μm sections for further management. Fibronectin was stained with anti-fibronectin polyclonal antibodies (Abcam), followed by FITC-labelled goat anti-rabbit IgG (H + L). Finally, the nuclei were stained with DAPI solution at room temperature for 10 min in the dark. Immunofluorescence images were obtained utilizing a Leica SP8 STED 3X system.

***In vivo* biosafety assay**

Five- to six-week-old female Balb/c mice (Vital River Laboratories, Beijing, China) were intravenously injected with 100 μL SPIOs@A-T, SPIOs@nA-T, SPIOs@A-nT NPs, and Vivotrax at a dose of 0.3 mg/mL Fe (n = 3). In addition, another group of mice was treated with the same volume of PBS as the control group (n = 3). After 48 h, all mice were euthanized, and their major organs (heart, liver, spleen, lung, kidney) and serum were collected for biosafety evaluation. Heart function biomarkers (high-density lipoprotein cholesterol, HDL-C), liver function indicators (alanine aminotransferase, ALT; aspartate aminotransferase, AST), and biochemical indicators of kidney function (blood urea nitrogen, BUN; creatinine, CREA) were examined using a blood biochemistry autoanalyzer (HITACHI 7600, Japan). The major organs were wax-embedded and sliced into 4-μm sections, followed by staining with haematoxylin and eosin (H&E). Images were captured using a PANNORAMIC MIDI II scanner (3DHISTECH, Hungary).

**Table. S1** The sequence of DNA aptamer used in this article

|  | Sequence of DNA aptamer (from 5 ' to 3 ') |
| --- | --- |
| Cy3-labelled ATP-responsive aptamer strand | Cy3-CAGTCACCTGGGGGAGTATTGCGGAGGAAGGT |
| Cy3-complementary quencher strand | TTTTTTTTTTTTTTTTTTTTTTTTTTTTTTTTTTTCCCAGGTGACTG-BHQ2 |
| Cy5.5-labelled ATP-responsive aptamer strand | Cy5.5-CAGTCACCTGGGGGAGTATTGCGGAGGAAGGT |
| Cy5.5-complementary quencher strand | triple SH-TTTTTTTTTTTTTTTTTTTTTTTTTTTTTTTTTTTCCCAGGTGACTG-BHQ2 |
| Cy5.5-labelled ATP-nonresponsive aptamer strand (mutated strand) | Cy5.5-CAGTCACCTGGGGGAGTATTGCAAAAACAGGT |


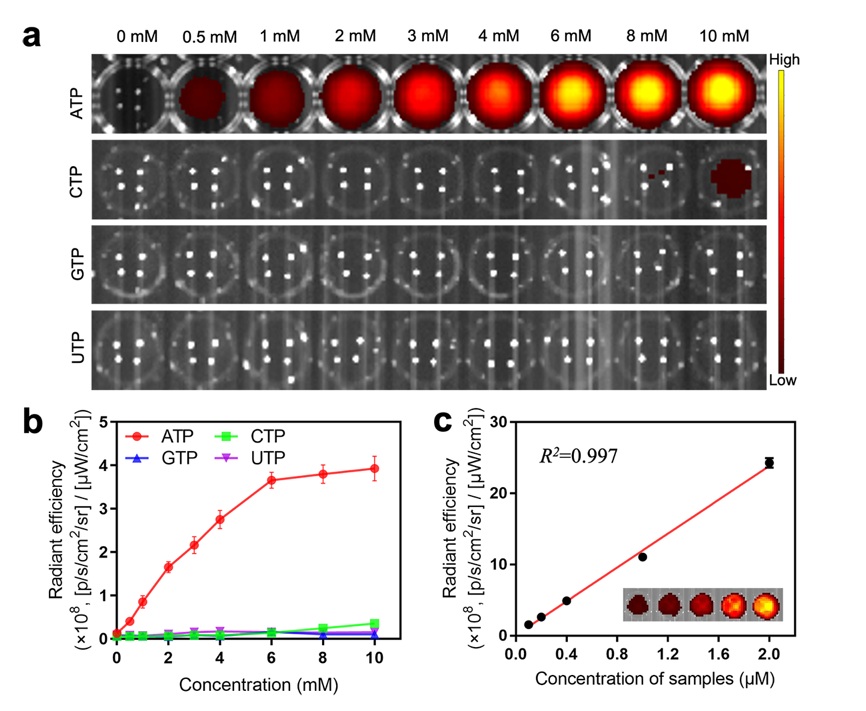


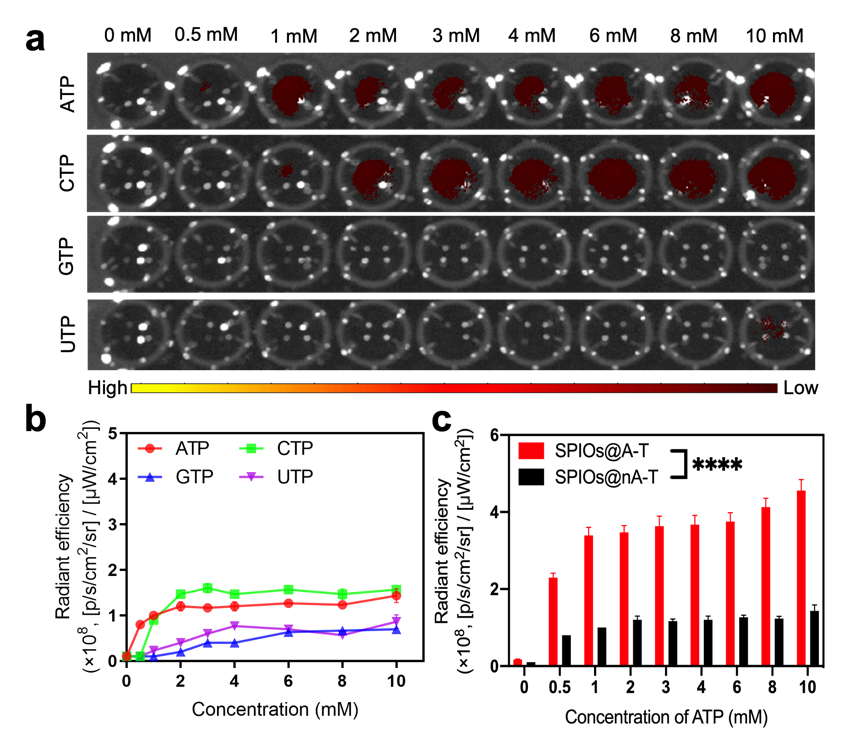
**Figure. S1** Fluorescence imaging properties of dsDNA-Cy3 probe. (a) Fluorescence imaging of the dsDNA-Cy3 probe responding to different nucleoside triphosphates, [Analytes]=100 nM. (b) Corresponding quantitative analysis of the dsDNA-Cy3 probe responding to different nucleoside triphosphates, [Analytes]= 100 nM. (c) Standard curve of the fluorescence signal over samples with series concentrations of dsDNA-Cy3 probe in response to 5 mM ATP.

Figure S2. Fluorescence imaging properties of SPIOs@nA-T NPs. Fluorescence imaging of SPIOs@nA-T NPs (a) and its corresponding quantitative analysis responding to different nucleoside triphosphates (b), [Analytes]=100 nM. (c) Comparison of fluorescence intensity between SPIOs@A-T NPs and SPIOs@nA-T NPs at different concentration of ATP. [Analytes]=100 nM, ****: *p* < 0.0001.


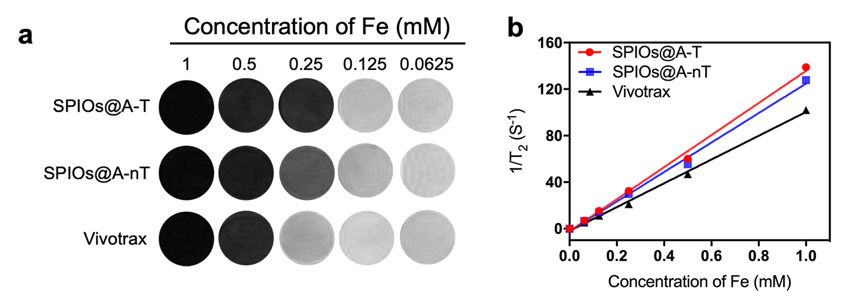


**
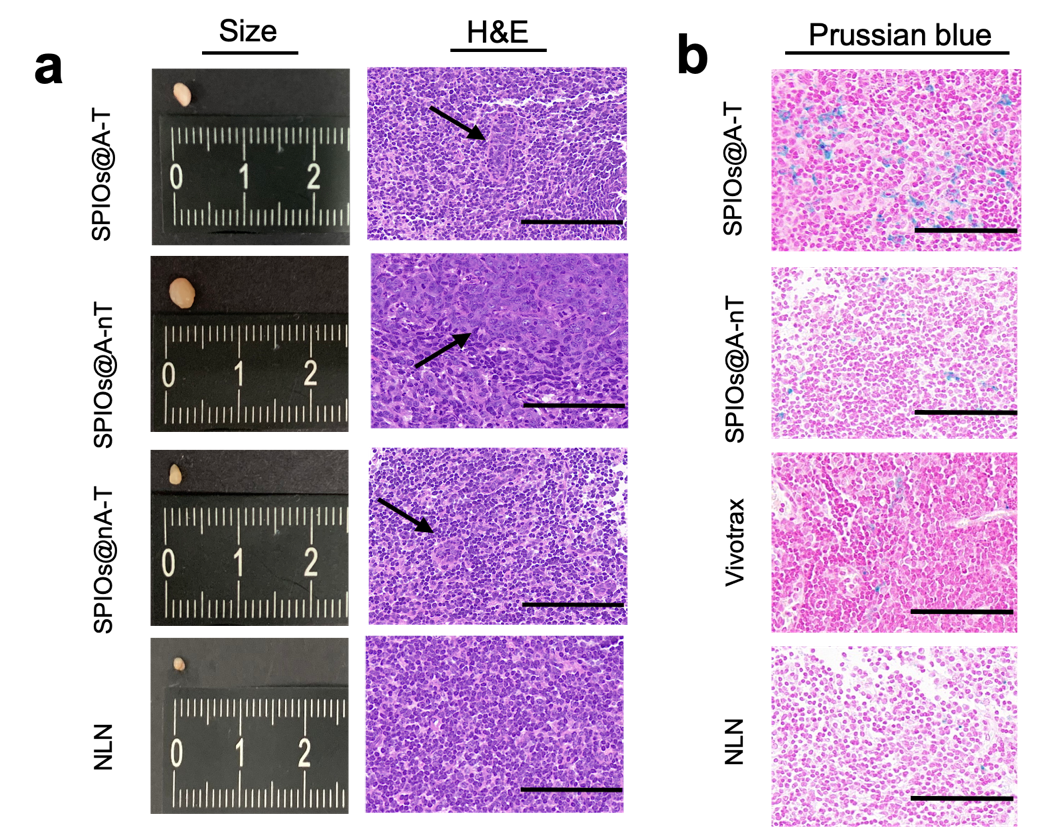
Figure. S3** Comparison of MRI imaging property. (a) MRI images of SPIOs@A-T, SPIOs@A-nT and Vivotrax

at a series of different concentrations. (b) Plot of and MRI signals of all samples.

**Figure. S4** Size and histopathological results of lymph nodes. (a) H&E staining images of MLN treated with SPIOs@A-T, SPIOs@nA-T and SPIOs@A-nT NPs, respectively. NLN treated with SPIOs@A-T NPs were seen as the control group. Black arrows: tumour cells in LN. (b) Prussian blue staining of MLN treated with SPIOs@A-T, SPIOs@A-nT and Vivotrax, respectively. NLN treated with SPIOs@A-T NPs was seen as the control group. Scale bars: 50 μm.


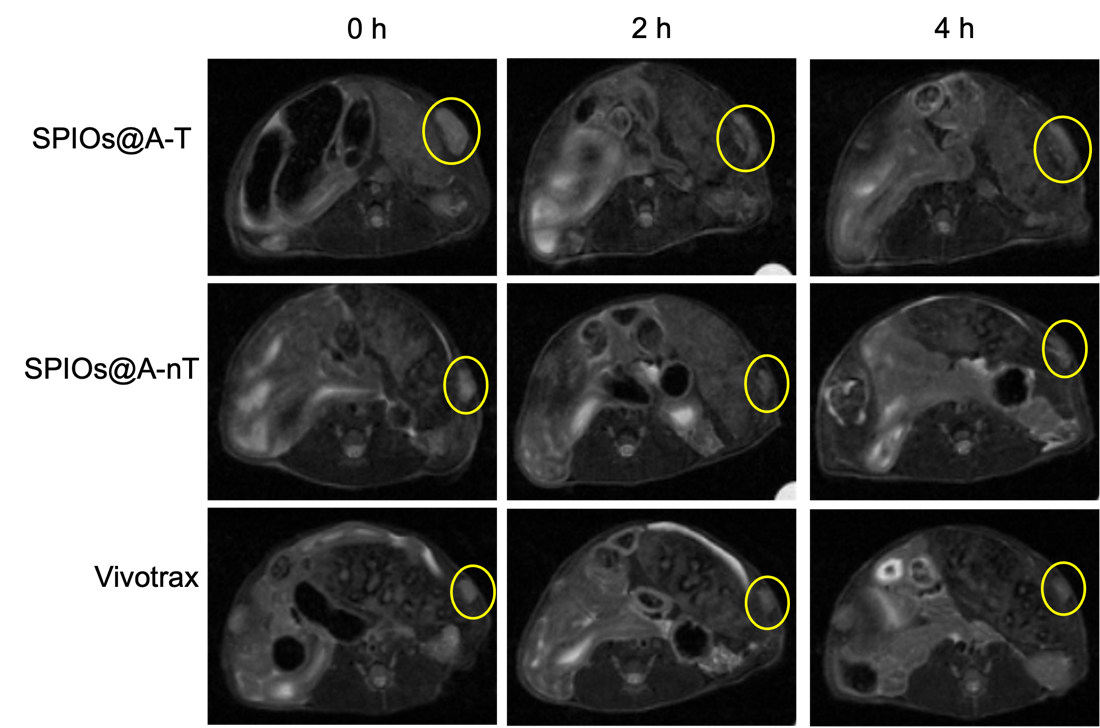


**Figure. S5** *In vivo* T2-weighted MRI images of MLN (yellow circle) treated with SPIOs@A-T, SPIOs@A-nT NPs and Vivotrax, respectively. Images were acquired at multiple timepoints (0, 2 and 4 h).


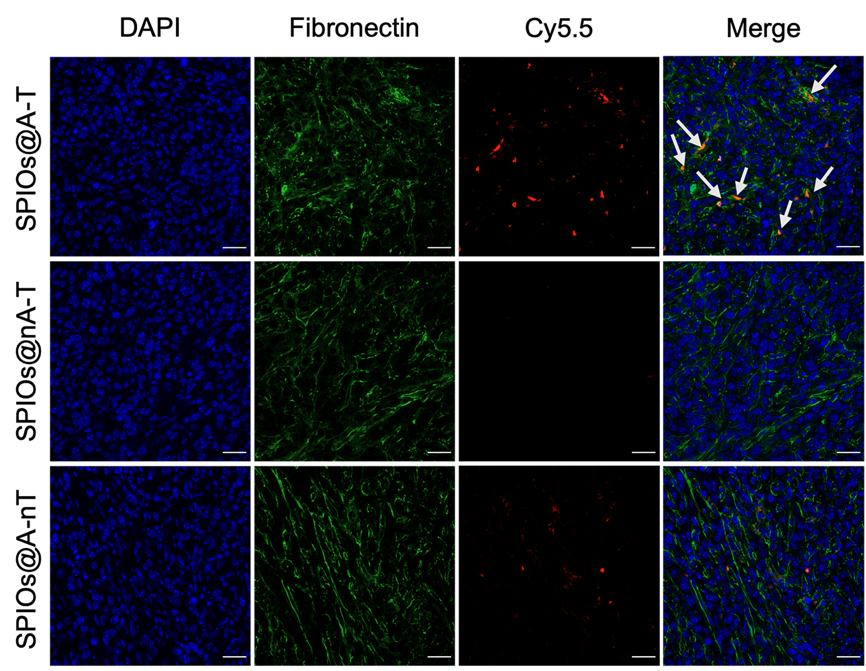


**Figure. S6** Immunofluorescence images of MLN treated with SPIOs@A-T, SPIOs@nA-T and SPIOs@A-nT NPs. DAPI: blue, fibronectin: green, SPIOs@A-T, SPIOs@nA-T and SPIOs@A-nT: red. Scale bars: 10 μm.


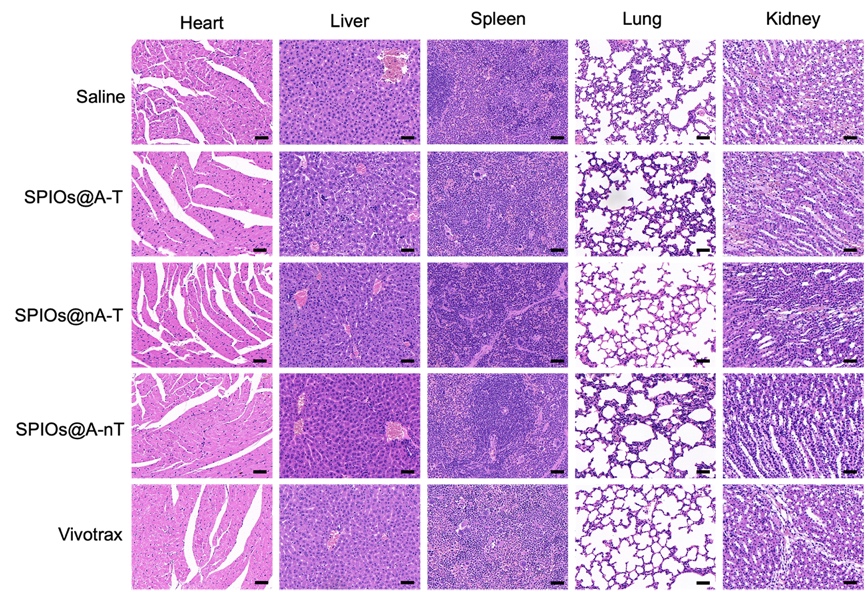


**Figure. S7** HE staining images of major organs (heart, liver, spleen, lung, and kidney) from different groups. Scale bars: 50 μm.


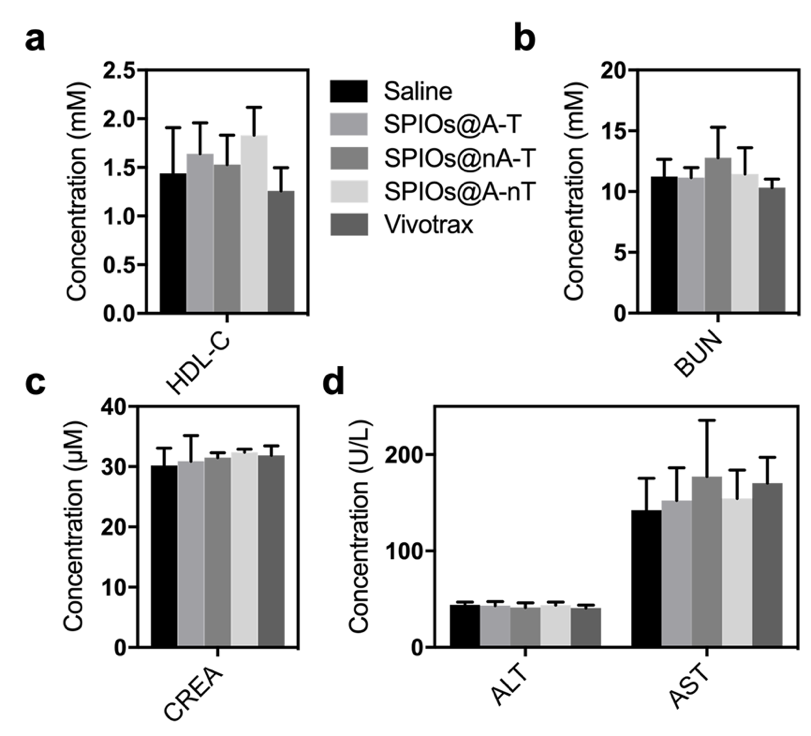


**Figure. S8** Blood biochemistry analysis of heart function biomarkers (HDL-C) (a), kidney function biomarkers (BUN and CREA) (b-c), and hepatic function biomarkers (ALT and AST) (d).
